# Rickettsiose invasive mortelle diagnostiquée tardivement en reanimation

**DOI:** 10.11604/pamj.2016.25.211.10452

**Published:** 2016-12-06

**Authors:** Hassen Ben Ghezala, Najla Feriani

**Affiliations:** 1 Réanimation Médicale à la Faculté de Médecine de Tunis, Hôpital de Zaghouan, Avenue de l’Environnement, 1100 Zaghouan, Tunisie; 2 Chirurgie Générale à la Faculté de Médecine de Tunis, Hôpital de Zaghouan, Avenue de l’Environnement, 1100 Zaghouan, Tunisie

**Keywords:** Rickettsiosis, Rickettsia Conorii, mortality, intensive care unit, Rickettsiosis, Rickettsia Conorii, mortality, Intensive Care Unit

## Abstract

La fièvre boutonneuse méditerranéenne est une maladie infectieuse du groupe des rickettsioses due à une bactérie intracellulaire: Rickettsia Conorii. Les formes pauci symptomatiques et bénignes sont prédominantes. Les formes graves sont rares et de plus en plus rapportées dans la littérature récente avec une atteinte multi systémique sévère pouvant menacer le pronostic vital. Nous rapportons dans ce travail un cas très rare d’un patient de 52 ans admis en réanimation pour convulsions, état de choc septique et insuffisance rénale aiguë. Devant la découverte au deuxième jour de la prise en charge de lésions de type « chancre d’inoculation » et boutonneuses, le diagnostic de rickettsiose grave avec une atteinte multi viscérale sévère a été évoqué puis confirmé par une sérologie. Une antibiothérapie par doxycycline a été alors introduite. Le patient va développer cependant une nécrose tubulaire aiguë nécessitant des séances d’épuration extra-rénale. Il va développer rapidement une défaillance multi viscérale mortelle. La rickettsiose, par la vascularite microcirculatoire qu’elle engendre, peut donner des tableaux aussi sévères. Les mécanismes impliqués et les facteurs pronostiques éventuels sont discutés dans ce travail à travers une revue de la littérature.

## Abstract (en)

Mediterranean spotted fever is an infectious disease belonging to the rickettsial group due to an intracellular bacterium: Rickettsia Conorii. Pauci-symptomatic and benign forms are predominant. Severe forms are rare and increasingly reported in the recent literature with potentially life-threatening severe multi-systemic involvement. We here report a very rare case of a 52-year old patient admitted to an Intensive Care Unit with convulsions, septic shock and acute renal failure. On the second day, given the discovery of « inoculation chancre »-like lesions which needed to be treated, the diagnosis of severe rickettsial disease with severe multivisceral involvement was suspected and then confirmed by serology. Antibiotic treatment with doxycycline was then administered. However the patient developed acute tubular necrosis requiring extrarenal purification sessions. The patient rapidly developed fatal multivisceral failure. Rickettsiosis is associated with microcirculatory vasculitis thus leading to severe clinical manifestations. The mechanisms involved and the possible prognostic factors have been discussed in this study through a review of the literature.

## Introduction

La rickettsiose est une infection répandue dans le monde et en Afrique. La fièvre boutonneuse méditerranéenne (FBM), qui en est sa forme méditerranéenne, est une infection due à une bactérie intra-cellulaire: *Rickettsia Conorii*. Elle est habituellement transmise par les tiques de chiens *Rhipicephalus sanguineus* [1] surtout pendant le printemps et l’été. Bien que la plupart des infections restent bénignes, près de 10 % des cas présentent des signes de gravité [2]. Nous rapportons dans ce travail un cas très rare d’une FBM compliquée d’une atteinte multiviscérale sévère: neurologique, hémodynamique et rénale chez un patient de 52 ans. Ces différentes complications associées au diagnostic tardif de la rickettsiose ont conduit au décès du patient en réanimation.

## Patient et observation

Un homme âgé de 52 ans a été hospitalisé en réanimation suite à une altération de l’état de conscience avec des crises convulsives subintrantes. Il a comme principal antécédent un diabète de type 2. Il a présenté brutalement une semaine avant son admission des convulsions tonico-cloniques généralisées dans un contexte de fièvre et d’altération de l’état général. Ces convulsions étaient suivies d’un déficit moteur avec une hémiplégie droite suivie d’une aphasie. Il a présenté également des douleurs abdominales diffuses et des vomissements abondants pour lesquelles il a eu une écographie abdominale montrant une hépatomégalie diffuse avec une flèche hépatique à 165 mm sans lésion focale décelable avec un foie d’échostructure homogène. Aucun signe associé n’était présent initialement. Il a été admis initialement dans le service de médecine interne. Il a eu un scanner cérébral revenu normal. Il a eu également un bilan biologique initial complet qui n’a pas montré d’anomalie en dehors de la cholestase. Devant les convulsions fébriles, une ponction lombaire a été pratiquée et était sans anomalies significatives.

Il a été transféré en réanimation secondairement devant l’altération persistante de son état de conscience. A son arrivée en réanimation, le patient va développer rapidement un syndrome de défaillance multi viscérale. Il a été intubé et ventilé puis mis sous antibiothérapie à large spectre (imipenème-vancomycine) et sous catécholamines (noradrénaline) devant l’apparition d’un état de choc septique. La recherche initiale d’un foyer infectieux était négative : radiographie thoracique, examen cytobactériologique des urines (ECBU) et hémocultures quotidiennes.

Le bilan hépatique pratiqué en réanimation avait montré une cholestase à prédominance conjuguée avec une bilirubine conjuguée à 41 UI/L sans cytolyse. Le bilan rénal a révélé une insuffisance rénale aiguë avec une créatinémie à 280 µmol/L. L’ionogramme sanguin a trouvé une hyponatrémie à 123 mmol/l. On a noté également un syndrome inflammatoire biologique avec une hyperleucocytose à prédominance de polynucléaires neutrophiles (Tableau 1). Une échographie cardiaque à la recherche d’une endocardite était négative.

**Tableau 1:** Bilan biologique du patient à l’admission en soins intensifs

Bilan Biologique	A l’admission
Urée (mmol/l) / Créatinémie (μmol/l)	24/280
Globules blancs (/mm^3^)	27600
Polynucléaires neutrophiles (/mm^3^)	18500
C-Réactive protéine (CRP)	152
Procalcitonine (μg/l)	2.3
Bilirubine totale/conjuguée (UI/L)	44.03/41.48
Natrémie (mmol/l)	123
Kaliémie/chlorémie (mmol/l)	4.7/99

**Figure 1:**
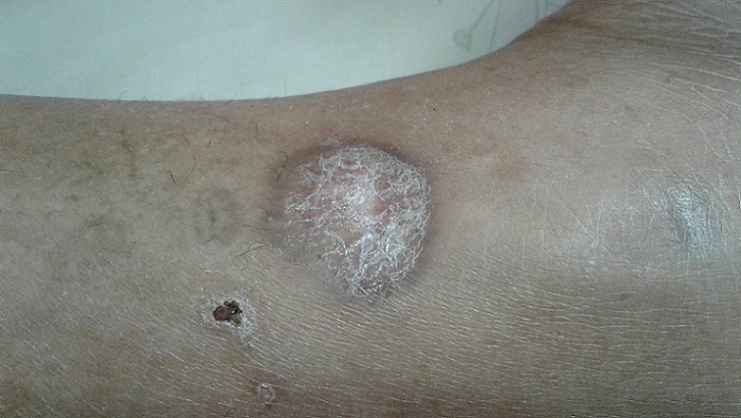
Jambe du patient montrant le chancre d’inoculation (tache noire) avec une lésion croûteuse

**Figure 2:**
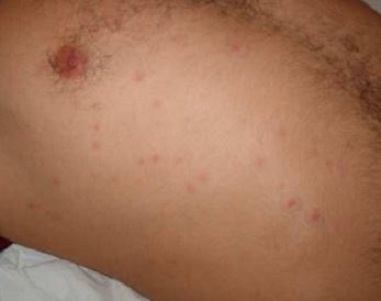
Tronc du patient montrant des lésions cutanées boutonneuses

Un examen attentif et spécialisé de la peau et des phanères au 2^ème^ jour, a remarqué la présence d’une tache noire au niveau de la face externe du tiers inférieur de la jambe droite au-dessus de la malléole externe droite accompagnée d’une escarre d’inoculation. Il s’agissait d’une lésion indolore, noirâtre et croûteuse, de 3 à 5 mm centrée sur un halo inflammatoire de 2 à 3 cm de diamètre, accompagnée d’une adénopathie (Figure 1). On a remarqué également l’apparition le même jour de plusieurs lésions avec un exanthème au niveau du tronc évoluant par poussées vers les membres, d’abord maculeuse puis papuleuse, finissant par donner l´aspect boutonneux (Figure 2). Le diagnostic de fièvre boutonneuse grave compliquée d’une défaillance multi viscérale : hémodynamique, neurologique et rénale a été alors évoqué. Les sérologies à la recherche de *Rickettsia Conorii* reviendront positives. Le patient a été mis alors sous traitement antibiotique par doxycycline à la dose de 200 mg par 24 heures.

Au troisième jour de la prise en charge en réanimation, le patient va aggraver l’insuffisance rénale aiguë sur nécrose tubulaire aiguë. Il va présenter une hyperkaliémie menaçante jusqu’à 8 mmol/l et des séances quotidiennes d’hémodialyse conventionnelle ont été indiquées et réalisées. Une échographie rénale pratiquée n’a pas montré de dilatation des cavités pyélo-calicielles ni de lésion focale décelable.

La patiente décède rapidement dans un tableau d’état de mort cérébrale avec engagement et coma dépassé.

## Discussion

La rickettsiose regroupe un grand nombre de maladies infectieuses dues au genre rickettsia. La FBM est sa forme méditerranéenne et a été rapportée dès 1910. La plupart des formes cliniques sont bénignes avec même des formes totalement asymptomatiques Les formes sévères avec une atteinte multi systémique restent rarement mais particulièrement sévères avec un taux de mortalité qui pourrait atteindre 35% [3]. Parmi les facteurs de risque de forme grave qui ont été rapportées, on retrouve un âge supérieur à 50 ans, l’alcoolisme, le tabagisme, l’insuffisance cardiorespiratoire, l’immunodépression et le diabète comme le cas chez notre patient. Par ailleurs, un diagnostic tardif avec un retard de la mise en route d’une antibiothérapie efficace à base de doxycycline a été identifié aussi comme un facteur indépendant de mauvais pronostic [4].

Il existe plusieurs hypothèses physiopathologiques expliquant les formes multi systémiques sévères. La plus probable est la vascularite associée à la rickettsiose avec une augmentation de la perméabilité capillaire micro vasculaire. L’hyperleucocytose et l’infiltration pulmonaire ont été aussi associées aux formes sévères de FBM. Dans la rickettsiose, les anomalies histopathologiques décrites sont surtout vasculaires, plus fréquentes au niveau cutané, musculaire, cardiaque, pulmonaire et cérébral. Ces lésions sont souvent disséminées le long des artères, veines et capillaires avec un processus inflammatoire irrégulier et la présence par endroits de congestion et d’œdème [5].

L’insuffisance rénale décrite chez notre patient serait très probablement due à une nécrose tubulaire aiguë faisant suite à une insuffisance rénale aiguë fonctionnelle sur une déshydratation précoce. En effet, la fièvre, les vomissements chez des patients souvent en état hémodynamique critique, expliquent la survenue rapide d’une nécrose tubulaire aiguë associée chez notre patient à une hyponatrémie qui atteste elle-aussi du volume des déperditions digestives. Quelques rares cas d’insuffisance rénale aiguë avec glomérulonéphrite extra-capillaire ont été rapportés [6]. De plus en plus des cas d’atteintes viscérales graves sont rapportés : syndrome de détresse respiratoire aiguë, myocardite, méningite, insuffisance hépatocellulaire, cytolyse ou même une quadriplégie sévère [7]. Même si la ponction lombaire était normale chez notre patient, nous pensons que les convulsions étaient dues à une atteinte endothéliale micro vasculaire cérébrale directe.

Les signes cutanés de la rickettsiose peuvent apparaitre tardivement comme c’est le cas chez notre patient et ainsi retarder la mise en route d’une antibiothérapie efficace. Ces patients sont souvent mis à tort sous antibiothérapie empirique par béta-lactamines ce qui aggrave leur pronostic. De plus, les sérologies de rickettsiose arrivent souvent tardivement ce qui retarde encore la mise en route d’un traitement efficace par les cyclines. Des nouvelles techniques de biologie moléculaire ou se basant sur l’examen anatomopathologique sont discutées dans la littérature afin de pallier à cet inconvénient [8].

Enfin, il faut se rappeler que les antibiotiques efficaces sur le groupe des rickettsioses sont les cyclines et les nouvelles quinolones essentiellement et non les pénicillines. Ceci explique souvent une évolution rapide mortelle due à une antibiothérapie tardive et inefficace comme chez notre patient [9].

## Conclusion

Il d’agit d’un cas exceptionnel de fièvre boutonneuse méditerranéenne compliquée d’une insuffisance rénale aiguë, d’un état de choc et de convulsions aggravés par un diagnostic tardif fatal lié à l’apparition tardive des signes cutanées et à une antibiothérapie retardée. En conclusion, bien que rare, il faut penser au diagnostic étiologique de rickettsiose devant un état de choc septique avec une porte d’entrée non identifiée et devant une atteinte multi viscérale évocatrice : hémodynamique, rénale, neurologique et hépatique.
